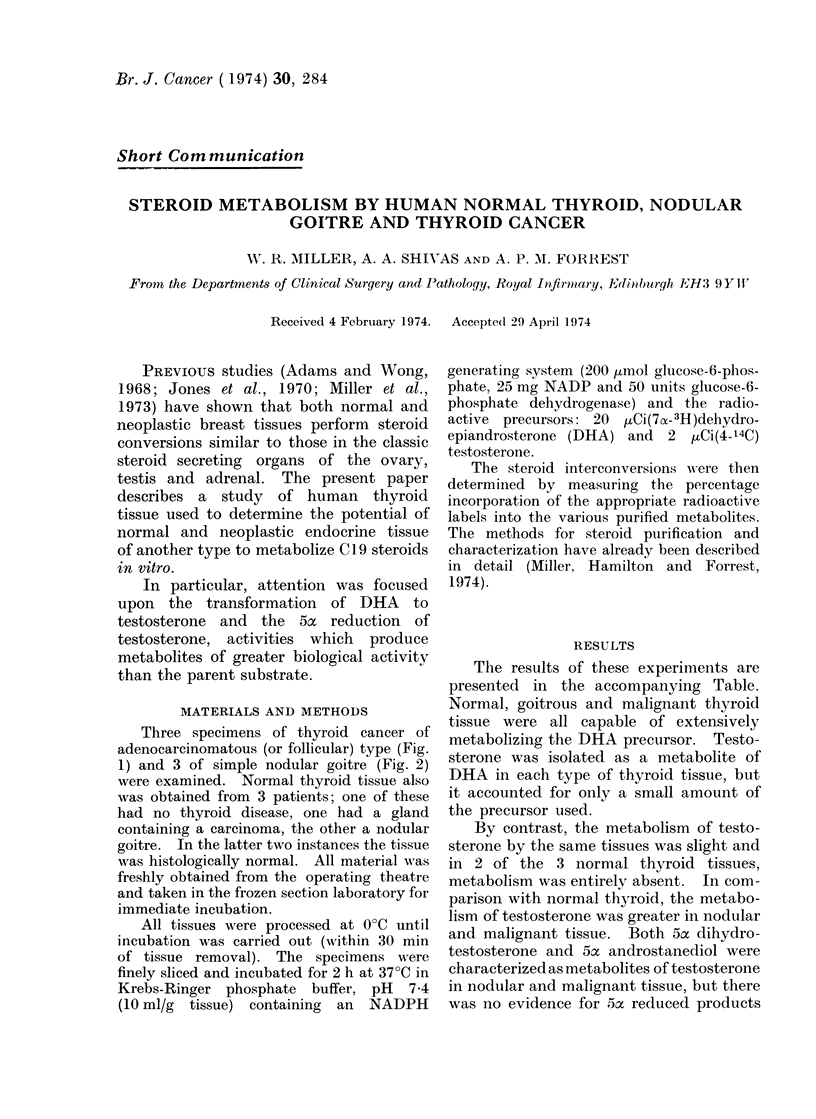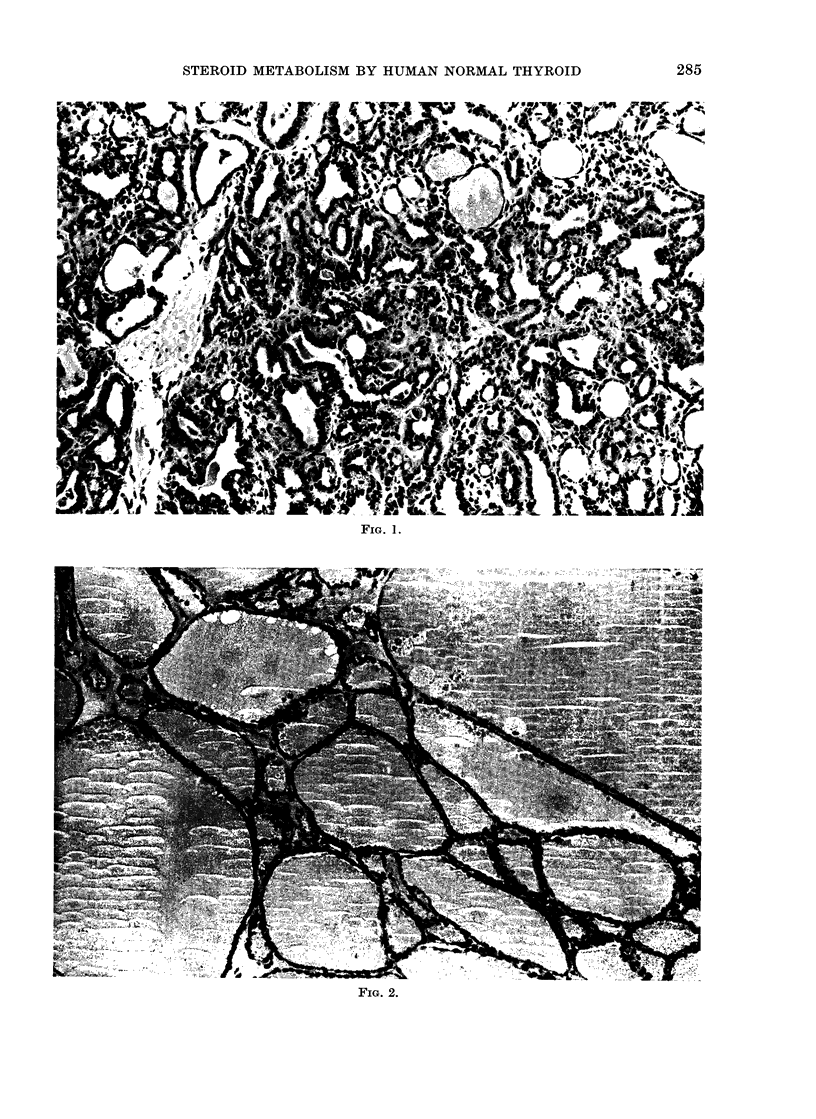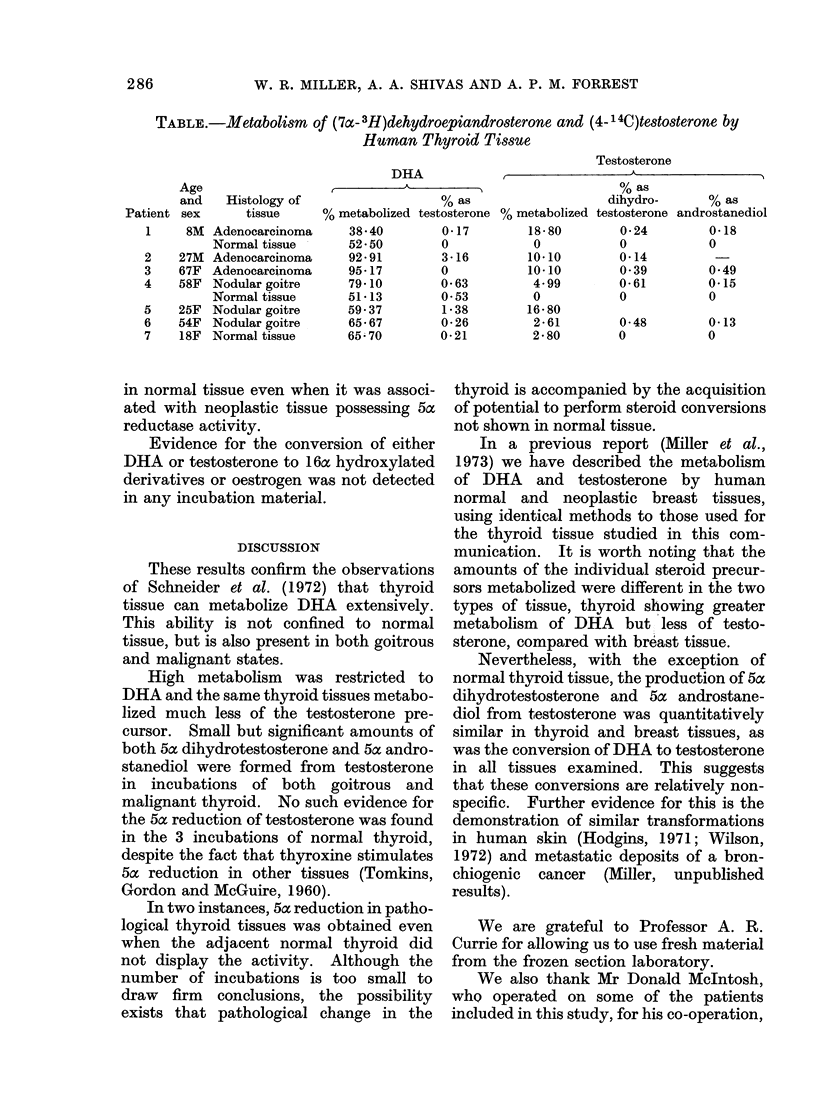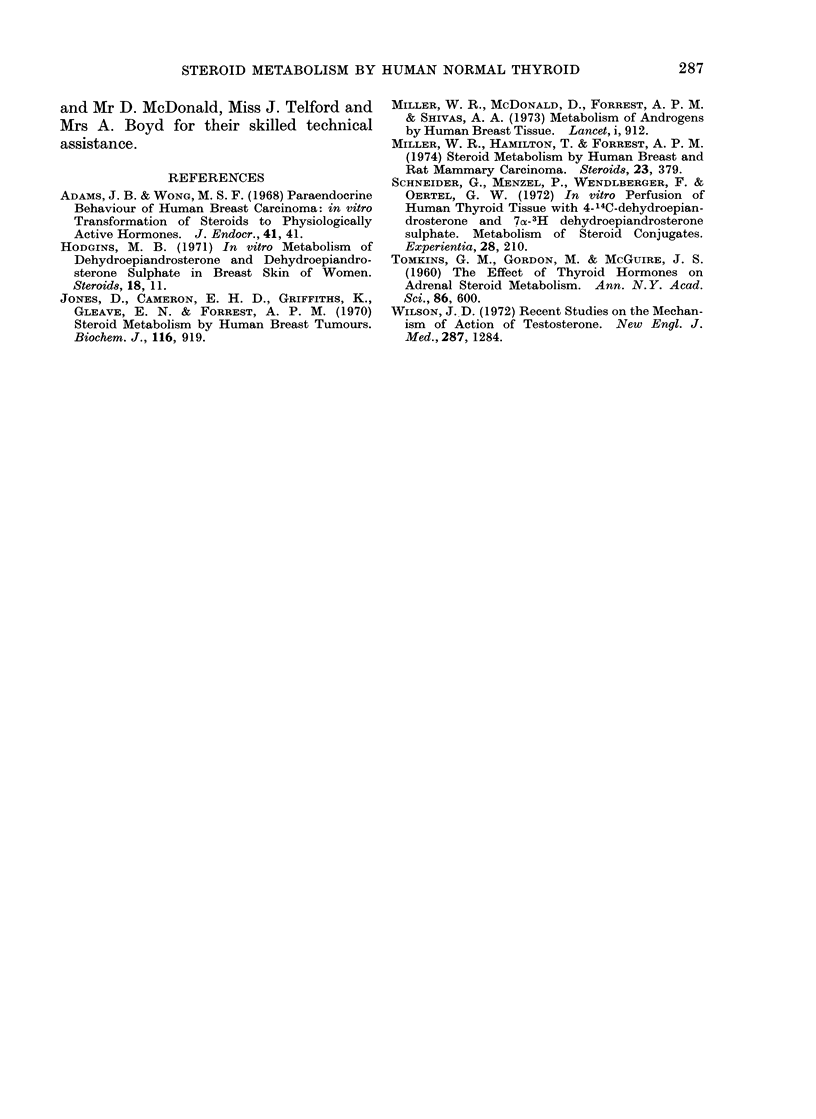# Steriod metabolism by human normal thyroid, nodular goitre and thyroid cancer.

**DOI:** 10.1038/bjc.1974.193

**Published:** 1974-09

**Authors:** W. R. Miller, A. A. Shivas, A. P. Forrest

## Abstract

**Images:**


Br. J. Cancer (1974) 30, 284

### *Short Communication*

### STEROID METABOLISM BY HUMAN NORMAL THYROID, NODULAR GOITRE AND THYROID CANCER

W. R. MILLER, A. A. SHIVAS AND A. P. M. FORREST

*From the Departments of Clinical Surgery and Pathology, Royal Infirmary, Edinburgh EH3 9YW*

Received 4 February 1974. Accepted 29 April 1974

PREVIOUS studies (Adams and Wong, 1968; Jones *et al.*, 1970; Miller *et al.*, 1973) have shown that both normal and neoplastic breast tissues perform steroid conversions similar to those in the classic steroid secreting organs of the ovary, testis and adrenal. The present paper describes a study of human thyroid tissue used to determine the potential of normal and neoplastic endocrine tissue of another type to metabolize C19 steroids *in vitro*.

In particular, attention was focused upon the transformation of DHA to testosterone and the 5α reduction of testosterone, activities which produce metabolites of greater biological activity than the parent substrate.

### MATERIALS AND METHODS

Three specimens of thyroid cancer of adenocarcinomatous (or follicular) type (Fig. 1) and 3 of simple nodular goitre (Fig. 2) were examined. Normal thyroid tissue also was obtained from 3 patients; one of these had no thyroid disease, one had a gland containing a carcinoma, the other a nodular goitre. In the latter two instances the tissue was histologically normal. All material was freshly obtained from the operating theatre and taken in the frozen section laboratory for immediate incubation.

All tissues were processed at 0°C until incubation was carried out (within 30 min of tissue removal). The specimens were finely sliced and incubated for 2 h at 37°C in Krebs-Ringer phosphate buffer, pH 7·4 (10 ml/g tissue) containing an NADPH generating system (200 μmol glucose-6-phosphate, 25 mg NADP and 50 units glucose-6-phosphate dehydrogenase) and the radioactive precursors: 20 μCi(7α-$^{3}$H)dehydroepiandrosterone (DHA) and 2 μCi(4-$^{14}$C) testosterone.

The steroid interconversions were then determined by measuring the percentage incorporation of the appropriate radioactive labels into the various purified metabolites. The methods for steroid purification and characterization have already been described in detail (Miller, Hamilton and Forrest, 1974).

### RESULTS

The results of these experiments are presented in the accompanying Table. Normal, goitrous and malignant thyroid tissue were all capable of extensively metabolizing the DHA precursor. Testosterone was isolated as a metabolite of DHA in each type of thyroid tissue, but it accounted for only a small amount of the precursor used.

By contrast, the metabolism of testosterone by the same tissues was slight and in 2 of the 3 normal thyroid tissues, metabolism was entirely absent. In comparison with normal thyroid, the metabolism of testosterone was greater in nodular and malignant tissue. Both 5α dihydrotestosterone and 5α androstanediol were characterized as metabolites of testosterone in nodular and malignant tissue, but there was no evidence for 5α reduced products

FIG. 1.

FIG. 2.

TABLE.—*Metabolism of* ($7\alpha$-$^{3}H$)*dehydroepiandrosterone and* (4-$^{14}$C)*testosterone by Human Thyroid Tissue*

| Patient | Age and sex | Histology of tissue | DHA % metabolized | DHA % as testosterone | Testosterone % metabolized | Testosterone % as dihydro-testosterone | Testosterone % as androstanediol |
|---|---|---|---|---|---|---|---|
| 1 | 8M | Adenocarcinoma | 38·40 | 0·17 | 18·80 | 0·24 | 0·18 |
| | | Normal tissue | 52·50 | 0 | 0 | 0 | 0 |
| 2 | 27M | Adenocarcinoma | 92·91 | 3·16 | 10·10 | 0·14 | — |
| 3 | 67F | Adenocarcinoma | 95·17 | 0 | 10·10 | 0·39 | 0·49 |
| 4 | 58F | Nodular goitre | 79·10 | 0·63 | 4·99 | 0·61 | 0·15 |
| | | Normal tissue | 51·13 | 0·53 | 0 | 0 | 0 |
| 5 | 25F | Nodular goitre | 59·37 | 1·38 | 16·80 | | |
| 6 | 54F | Nodular goitre | 65·67 | 0·26 | 2·61 | 0·48 | 0·13 |
| 7 | 18F | Normal tissue | 65·70 | 0·21 | 2·80 | 0 | 0 |

in normal tissue even when it was associated with neoplastic tissue possessing $5\alpha$ reductase activity.

Evidence for the conversion of either DHA or testosterone to $16\alpha$ hydroxylated derivatives or oestrogen was not detected in any incubation material.

DISCUSSION

These results confirm the observations of Schneider *et al.* (1972) that thyroid tissue can metabolize DHA extensively. This ability is not confined to normal tissue, but is also present in both goitrous and malignant states.

High metabolism was restricted to DHA and the same thyroid tissues metabolized much less of the testosterone precursor. Small but significant amounts of both $5\alpha$ dihydrotestosterone and $5\alpha$ androstanediol were formed from testosterone in incubations of both goitrous and malignant thyroid. No such evidence for the $5\alpha$ reduction of testosterone was found in the 3 incubations of normal thyroid, despite the fact that thyroxine stimulates $5\alpha$ reduction in other tissues (Tomkins, Gordon and McGuire, 1960).

In two instances, $5\alpha$ reduction in pathological thyroid tissues was obtained even when the adjacent normal thyroid did not display the activity. Although the number of incubations is too small to draw firm conclusions, the possibility exists that pathological change in the thyroid is accompanied by the acquisition of potential to perform steroid conversions not shown in normal tissue.

In a previous report (Miller *et al.*, 1973) we have described the metabolism of DHA and testosterone by human normal and neoplastic breast tissues, using identical methods to those used for the thyroid tissue studied in this communication. It is worth noting that the amounts of the individual steroid precursors metabolized were different in the two types of tissue, thyroid showing greater metabolism of DHA but less of testosterone, compared with breast tissue.

Nevertheless, with the exception of normal thyroid tissue, the production of $5\alpha$ dihydrotestosterone and $5\alpha$ androstanediol from testosterone was quantitatively similar in thyroid and breast tissues, as was the conversion of DHA to testosterone in all tissues examined. This suggests that these conversions are relatively nonspecific. Further evidence for this is the demonstration of similar transformations in human skin (Hodgins, 1971; Wilson, 1972) and metastatic deposits of a bronchiogenic cancer (Miller, unpublished results).

We are grateful to Professor A. R. Currie for allowing us to use fresh material from the frozen section laboratory.

We also thank Mr Donald McIntosh, who operated on some of the patients included in this study, for his co-operation, and Mr D. McDonald, Miss J. Telford and Mrs A. Boyd for their skilled technical assistance.

### REFERENCES

ADAMS, J. B. & WONG, M. S. F. (1968) Paraendocrine Behaviour of Human Breast Carcinoma: *in vitro* Transformation of Steroids to Physiologically Active Hormones. *J. Endocr.*, **41**, 41.

HODGINS, M. B. (1971) *In vitro* Metabolism of Dehydroepiandrosterone and Dehydroepiandrosterone Sulphate in Breast Skin of Women. *Steroids*, **18**, 11.

JONES, D., CAMERON, E. H. D., GRIFFITHS, K., GLEAVE, E. N. & FORREST, A. P. M. (1970) Steroid Metabolism by Human Breast Tumours. *Biochem. J.*, **116**, 919.

MILLER, W. R., MCDONALD, D., FORREST, A. P. M. & SHIVAS, A. A. (1973) Metabolism of Androgens by Human Breast Tissue. *Lancet*, i, 912.

MILLER, W. R., HAMILTON, T. & FORREST, A. P. M. (1974) Steroid Metabolism by Human Breast and Rat Mammary Carcinoma. *Steroids*, **23**, 379.

SCHNEIDER, G., MENZEL, P., WENDLBERGER, F. & OERTEL, G. W. (1972) *In vitro* Perfusion of Human Thyroid Tissue with 4-$^{14}$C-dehydroepiandrosterone and 7$\alpha$-$^{3}$H dehydroepiandrosterone sulphate. Metabolism of Steroid Conjugates. *Experientia*, **28**, 210.

TOMKINS, G. M., GORDON, M. & MCGUIRE, J. S. (1960) The Effect of Thyroid Hormones on Adrenal Steroid Metabolism. *Ann. N.Y. Acad. Sci.*, **86**, 600.

WILSON, J. D. (1972) Recent Studies on the Mechanism of Action of Testosterone. *New Engl. J. Med.*, **287**, 1284.